# The abnormal umbilical venous–arterial index in the second half of pregnancy is associated with fetal outcome: A retrospective cross-sectional study

**DOI:** 10.3389/fped.2023.1036359

**Published:** 2023-03-10

**Authors:** Ling Wang, Dan Zhou, Baiguo Long, Jiqing Wang, Lingling Li, Yang Peng, Qichang Zhou, Shi Zeng

**Affiliations:** ^1^ Department of Ultrasound, Women and Children Healthcare Hospital of Zhu Zhou, Zhuzhou, China; ^2^ Department of Ultrasound Diagnosis, The Second Xiangya Hospital of Central South University, Changsha, China; ^3^ Department of Ultrasound, Women and Children Healthcare Hospital of Changsha, Changsha, China

**Keywords:** umbilical vein blood flow volume, umbilical artery pulsatility index, normalized umbilical vein blood flow volume, venous-arterial index, fetal outcome

## Abstract

**Objective:**

This study aims to observe the changes of the umbilical venous–arterial index (VAI) and investigate its predictive power for fetal outcome during the second half of pregnancy.

**Methods:**

Fetuses with gestational age (GA) at 24–39 weeks were collected. According to the outcome score, neonates with outcome scores of 0, 1, or 2 were assigned to the control group, whereas those with scores of 3–12 were assigned to the compromised group. VAI was calculated as the ratio of normalized umbilical vein blood flow volume and umbilical artery pulsatility index. Regression analysis was performed to obtain the best-fitting curves between VAI and GA in the controls. Doppler parameters and perinatal outcomes were compared in both groups. Receiver operating characteristic analysis was used to assess the diagnostic performance of the VAI.

**Results:**

A total of 833 (95%) fetuses had Doppler parameters and pregnancy outcomes documented. Compared with the controls, the VAI was significantly lower in the compromised group (83.2 vs. 184.8 ml/min/kg, *p* < 0.001). The sensitivity and specificity of VAI to predict compromised neonates were 95.15% (95% Cl, 89.14 to 97.91%) and 99.04% (95% CI: 98.03 to 99.53%), respectively at a cutoff value of 120 ml/min/kg.

**Conclusions:**

VAI presents better diagnostic performance than umbilical vein blood flow volume and umbilical artery pulsatility index. A cutoff value of 120 ml/min/kg might be used as the warning value for predicting the fetal outcome.

## Introduction

The umbilical cord usually consists of two umbilical arteries and a single umbilical vein. The human fetus receives all its oxygen and metabolic substrates from the placenta through the umbilical vein. Umbilical venous measurement has remained largely restricted to experimental studies and has not yet been integrated into routine clinical obstetric practice. Researchers have found that umbilical venous blood flow is reduced in intrauterine growth restriction (IUGR) fetuses (1–4). A previous study demonstrated that fetoplacental blood volume flow can be calculated directly from the umbilical vein and may provide a better index of placental perfusion than the umbilical artery velocity ratio (5). A progressive reduction in umbilical vein blood flow over time in IUGR fetuses was found even with normal umbilical artery Doppler parameters (1). The umbilical artery transports carbon dioxide and metabolite from fetus to the placenta. Umbilical artery blood flow is an important parameter for downstream impedance for fetal–placental perfusion. Umbilical artery pulsatility index (UAPI) increases with increasing placental vascular disease and it has been reported to be useful for the prediction of early-onset pregnancy-induced hypertension (6). However, a single umbilical artery index has difficulty accurately reflecting fetal conditions within the uterine environment. Tchirikov et al. proposed the umbilical venous–arterial index (VAI) as a predictor of neonatal compromise (7). They normalized umbilical venous blood flow by dividing the absolute flow by the estimated fetal weight expressed in kilograms. VAI is a dual hemodynamic index that includes both normalized umbilical venous blood flow volume (nQuv) and UAPI (7). Since blood flow is always related to the size of organs and tissues supplied, the ratio of nQuv to UAPI delineates the influence of fetal weight (4). Atabay et al. demonstrated that the VAI was more sensitive than the UV indices alone for fetal distress and had a moderate sensitivity and high negative predictive value for intrapartum fetal distress prediction (8). This study intends to observe the changes of umbilical venous–arterial index (VAI) and to investigate the predictive power of VAI for perinatal outcomes in the second half of pregnancy.

## Materials and methods

A retrospective, cross-sectional study was conducted between March 2018, and December 2020 at the Second Xiangya Hospital and Zhuzhou Maternal and Child Health Hospital in China. The study population included fetuses that were 24–39 weeks of gestation. The inclusion criteria were singleton pregnancy with an accurate gestational age (GA) based on the last menstrual period or verified by the crown-rump length during the first trimester. attending our hospital for antenatal care, or referring for obstetric indications. The exclusion criteria were multiple pregnancy, GA less than 24 weeks; presence of structural or chromosomal defects, and presence of maternal smoking history. This study was approved by the Ethics Committee of the Second Xiangya Hospital and Women and Children Healthcare Hospital of Zhu Zhou (2018-Yan070). Written informed consent was obtained from each pregnant woman.

**Figure 1:**
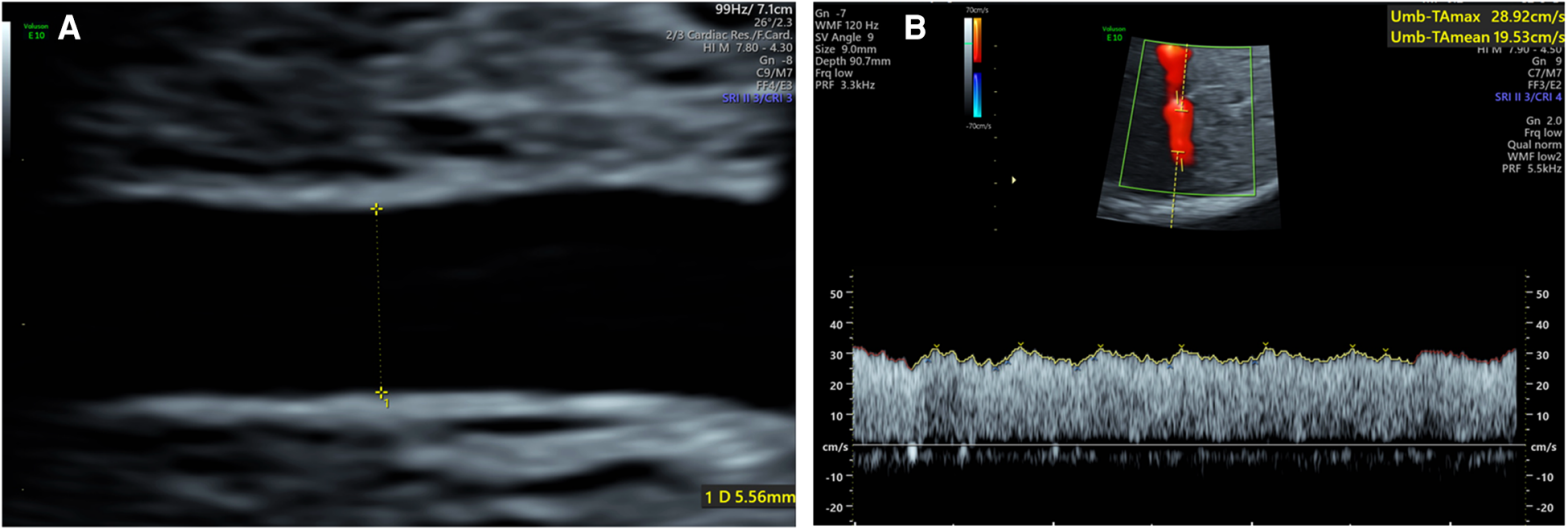
(**A**) Umbilical vein diameter measurement of the intra-abdominal portion of a 34 weeks fetus; (**B**) umbilical vein vmax measurement of a 32 weeks fetus.

All ultrasound examinations were completed by a single examiner (LW). Ultrasound measurements were performed using Voluson E10 (GE Healthcare Ultrasound, USA) ultrasound system with a C2-9-D probe and Voluson E8 (GE Healthcare Ultrasound, USA) ultrasound system with a C1-5-D probe. Fetal biometry, including measurements of the biparietal diameter, head circumference, femur length and abdominal circumference, was performed. Fetal weight (EFW) was estimated automatically using software associated with the ultrasonic instrument. For the umbilical artery measurement, a free segment of the umbilical artery was selected. The image was magnified to at least 30% of the screen. The sample volume was equivalent to the width of the vessel and the Doppler angle was kept below 15°. The UAPI was recorded by automated waveform tracing. For umbilical vein measurements, a straight intra-abdominal portion located distal to the ductus venosus and the portal branch was selected. Both the time averaged maximum velocity (Vmax) and umbilical vein diameter (Duv) were measured in longitudinal image plane following the “maximum principle” (7) (1). The vessel walls on screen should appear as straight lines for as long as possible (maximum length); (2). The vessel diameter should be maximal; (3). The Vmax should be the maximum value. The maximum inner diameter was measured perpendicularly from the inner edge to the inner edge across the vessel lumen when the image was magnified to at least 30% of the screen (Figure 1A). To obtain the maximum velocity, the ultrasound probe needed to be tilted by 90° (9, 10). Adjust the high-pass wall filter to the lowest and the scale to 10–15 cm/s. The Doppler sample volume was positioned as close to the vein diameter as possible. When the vessel was large (>32 weeks of gestation), a Doppler gate was positioned to cover one side of the inner wall and the center of the vein (11) (Figure 1B). The insonation angle was maintained below 15°. Automated waveform tracing was used to envelope the waveforms. All Doppler evaluations were carried out in the absence of fetal breathing and body movement. Each measurement was repeated three times, and the mean was used for further analyses. We used the following formula to calculate the blood flow volume in the umbilical vein (Quv, ml/min) (12, 13): Quv = *π* (*D*/2)^2^0.5Vmax × 60. As the Quv is proportional to the fetal weight, umbilical venous blood flow volume was normalized as ml/min/kg (nQuv = Quv/EFW) (12, 13). The nQuv/UAPI ratio was calculated as VAI (7).

Details regarding the pregnancy process and outcomes, including duration of gestation, mode of delivery, birth weight, Apgar score, and umbilical cord blood gas analysis were recorded. All the fetuses were followed up clinically in the same way and that there were no differences among doctors on duty on the indications to delivery or prolong pregnancy. The delivery management of fetuses of small for gestational age was according to guidelines for clinical practice and recommendation of the Chinese society of Obstetrics and Gynecology. For fetus > 37 weeks of gestation, active termination of pregnancy may be considered. For fetus at 34–37 weeks of gestation, a single increase in umbilical artery Doppler flow is not used as an indication of immediate labor. The systematic evaluation of fetal health is considered and the changes should be followed up closely. If the fetus is well monitored, delivery can be expected after 37 weeks. Active termination of pregnancy may be considered for fetuses > 34 weeks if they show stagnant growth > 2 weeks, oligohydramnios (maximum depth <2 cm), biophysical profile (BPP) < 6, frequent abnormal patterns in non-stress tests (NST), or definite Doppler flow abnormalities. For fetus at 32–34 weeks of gestation, if umbilical artery end-diastolic blood flow is lack and there is no other evidence of fetal distress (such as abnormal pattern of intrapartum cardiotocograph (CTG), BPP < 4, abnormal ductus venosus waveform (A wave), pregnancy may be expected to 34 weeks of gestation. For 28–32 weeks of gestation, if there is abnormal umbilical artery blood flow (end-diastolic blood flow is absent or reversed) combined with abnormal A wave in ductus venosus (absent or reversed), active termination of pregnancy is considered after the completion of corticosteroids to promote fetal lung maturation as soon as possible. In the case of single end-diastolic reversal of umbilical artery flow in the absence of other evidence of fetal distress (such as abnormal CTG pattern, abnormal ductus venosus waveform), pregnancy may be delayed until no more than 32 weeks of gestation. All neonates were evaluated by a pediatrician. Basic score values of 0, 1, or 2 were assigned to each of six outcome variables (umbilical arterial blood pH, Apgar score after 1 min, birth weight, duration of gestation, type of respiratory support, and admission to the pediatric department), and the basic score values were summed to obtain an “outcome score” (Table 1). According to the outcome score, neonates with outcome scores of 0, 1, or 2 were assigned to the control group, whereas those with scores of 3–12 were assigned to the compromised group (7).

**Table 1:** Score parameters for evaluating fetal outcome (7).

	0	1	2
PH (cord blood)	≥7.2	<7.2 or ≥7.1	<7.1
Apgar score at 1 min	≥8	6–7	≤5
Birth weight (percentile)	≥10th	<10th and ≥3rd	<3rd
Duration of gestation (weeks)	≥37	32–37	<32
Respiratory support	None	Mask	Intubation
Transfer	None	Neonatal department	Intensive care unit

The results are presented as the median (range) for continuous variables and as frequencies for categorical variables. The Shapiro-Wilk W test was performed to assess the normality of the data distribution. Scatter diagrams were shown to demonstrate the relation between Duv, Vmax, Quv, nQuv, UAPI, VAI and GA. Data were compared between the controls and compromised group using Student's *t*-test or the Mann–Whitney *U* test for continuous variables and chi-square test or Fisher's exact test for categorical variables. Receiver operating characteristic (ROC) analysis was used to assess the diagnostic performance of the VAI. Reproducibility analyses with interclass correlation coefficient and intraclass correlation coefficient (ICC) were performed. To assess interobserver reproducibility, 50 subjects were randomly selected from the pregnant women. The umbilical cord parameters were independently measured by a second reader, who was blinded to the clinical data of the cases. To assess intraobserver reproducibility, a single operator measured the values of these 50 subjects twice over a period of 2 h. *p* < 0.05 was considered statistically significant. All statistical analyses were performed with SPSS statistics software 20.0, IBM and GraphPad Prism 8.

## Results

Eighty hundred and seventy-six fetuses were initially included in this study. However, 32 fetuses did not have high-quality images or failed to meet the “maximum principle” for measurement, and 11 fetuses were lost to follow up. A total of 833 (95%) fetuses had documentation of Doppler parameters and pregnancy outcomes. According to the outcome score, the control group consisted of 730 fetuses and the compromised group contained 103 fetuses, 2 of which died within 24 h after birth (Supplementary Figure S1). The median outcome score was 0 (range 0–1) in the controls and 5 (range 4–6) in the compromised group. The interval between obtaining the measurement and delivery in the control and compromised groups were 63 days (95% CI, 60–65) and 33 days (95% CI, 28–38), respectively. The proportion of spontaneous delivery in controls was significantly higher than those in the compromised group (*p* < 0.001). Birth weight, duration of pregnancy, umbilical arterial blood pH and Apgar score after 1 min were significant lower (*p* < 0.05) and the proportion of respiratory support and admission to the pediatric department was significant higher in the compromised group (*p* < 0.05) (Table 2).

**Table 2:** Clinical and Doppler parameters of control fetuses and fetuses with compromised outcome.

Parameters	Control group *n* = 730	compromised group *n* = 103	*p* value
Maternal age, years	28 (24–35)	29(25–33)	0.61
Nulliparous, *n*	490(67.1%)	66 (64.1%)	0.539
GA at scan, weeks	32 (28–36)	31 (29–32)	0.001
EFW, g	1,915 (1160–2750)	1,509 (1030–1730)	<0.001
**Ultrasound**			
Duv, cm	0.59 (0.51–0.66)	0.54 (0.45–0.60)	<0.001
Vmax, cm/s	33.6 (28.8–38.3)	17.5 (15.3–19.4)	<0.001
Quv, ml/min	271.2(188.9–361.3)	121.5 (70.9–149.4)	<0.001
nQuv, ml/min/kg	148.6 (131.8–167.8)	83.3 (71.0–97.5)	<0.001
UAPI	0.8 (0.7–0.9)	1.0 (0.9–1.1)	<0.001
VAI, ml/min/kg	184.8 (163.6–201.4)	83.2 (73.6–97.6)	<0.001
**Fetal outcome**			
GA at delivery, weeks	39 (38–40)	36 (32–38)	<0.001
Birth weight, g	3,256 (2956–3433)	2,300 (1800–2800)	<0.001
Cord PH	7.29 (7.23–7.32)	7.17 (7.13–7.23)	0.006
Apgar score at 1 min	10 (9–10)	9 (8–10)	<0.001
**Respiratory support**			
Mask, *n*	32 (4.4%)	63 (61.2%)	<0.001
Intubation, *n*	0 (0.0%)	33 (32.0%)	<0.001
**Transfer**			
Neonatal department, *n*	53 (7.3%)	16 (15.5%)	0.004
Intensive care unit, *n*	0 (0.0%)	87 (84.5%)	<0.001
Neonatal death, *n*	0 (0.0%)	2 (1.9%)	0.015
Spontaneous delivery, *n*	610 (83.6%)	10 (9.7%)	<0.001

EFW, estimated fetal weight; Duv, diameter of umbilical vein; Quv, umbilical vein volume blood flow; nQuv, normalized umbilical vein blood flow volume; UAPI, umbilical artery pulsatility index; VAI, venous–arterial index.

**Figure 2:**
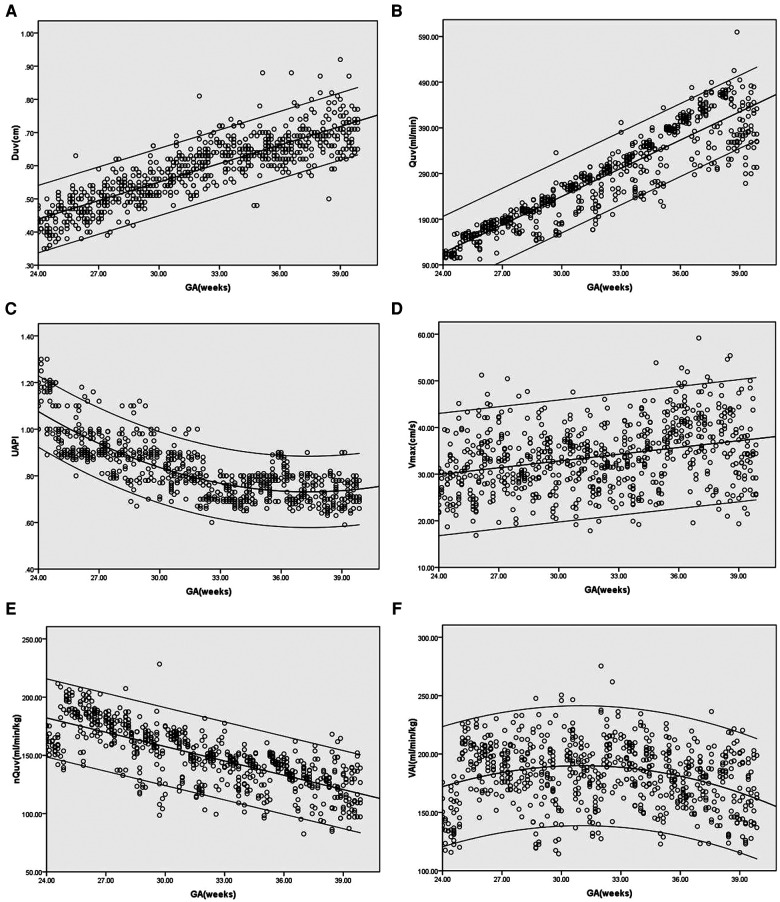
(**A–F**) Scatter diagrams of umbilical vein diameter (Duv), umbilical vein blood flow volume (Quv), umbilical artery pulsatility index (UAPI), vmax, normalized umbilical vein blood flow volume(nQuv), venous–arterial index (VAI) based on gestational age (GA). Mean and 5th and 95th percentiles are showed.

In the control group, Duv, Vmax, Quv and nQuv increased linearly with GA (*R*^2 ^= 0.73, 0.1, 0.84 and 0.55, respectively, *p* < 0.001), while UAPI declined with GA (*R*^2 ^= 0.64, *p* < 0.001). For VAI, a quadratic curve fit well (*R*^2 ^= 0.08, *p* < 0.001). VAI grew during 24 and 32 weeks of gestation and declined gradually after 32 weeks of gestation (Figure 2).

**Figure 3:**
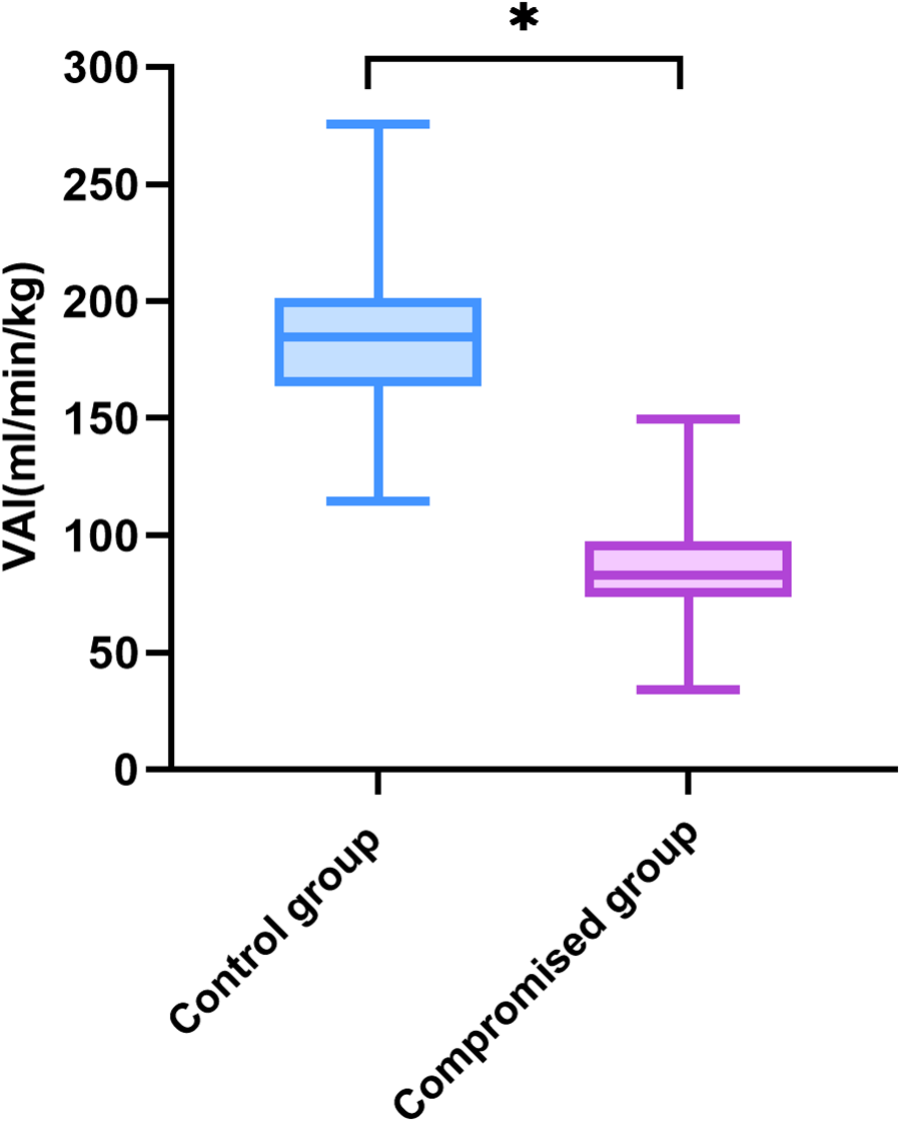
Venous–arterial index in the controls (*n* = 730) and the compromised fetuses (*n* = 103). Compared with the control group, *p < 0.001. VAI, venous–arterial index.

**Figure 4:**
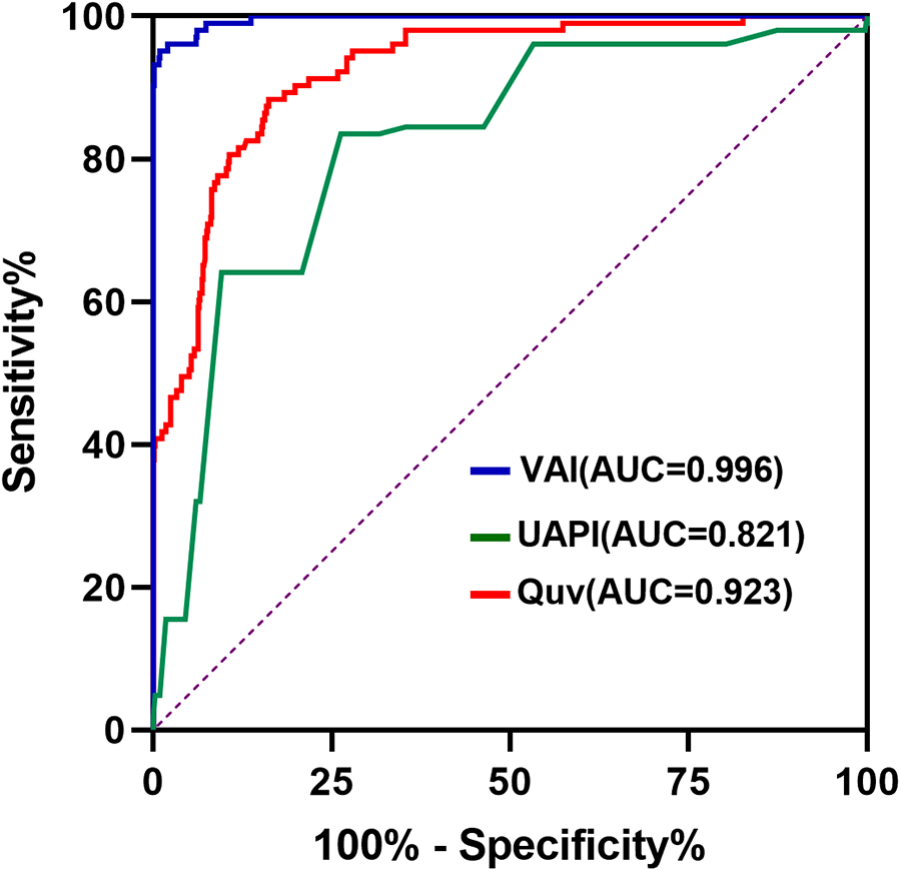
Receiver-operating characteristics curve of venous–arterial index (VAI), umbilical vein blood flow volume (Quv) and umbilical artery pulsatility index (UAPI) in the control and compromised groups. The cutoff value of VAI is 120 ml/min/kg.

Compared with controls, the Duv, Vmax, Quv, nQuv and VAI significantly decreased in the compromised group, while UAPI increased obviously (*p* < 0.001) (Table 2, Figure 3). ROC analysis showed that at a cutoff value of 120 ml/min/kg, the sensitivity and specificity of VAI in predicting compromised neonates were 95.15% (95% Cl, 89.14 to 97.91%) and 99.04% (95% CI: 98.03 to 99.53%), respectively. The area under receiver operating characteristic curve was 0.996 (95% Cl, 0.993–0.999; *p* < 0.001), which was higher than that of Quv (AUC:0.923, 95% Cl: 0.898–0.949; *p* < 0.001) and UAPI (AUC:0.821, 95% Cl: 0.778–0.865; *p* < 0.001) (Figure 4).

The compromised group was further divided into two groups according to the birth weight percentile. Compared with the fetuses of birth weight ≥10th percentile, the Quv, Vmax and VAI significant reduced in the fetuses of birth weight <10th percentile (*p* < 0.05), while the UAPI significantly elevated (*p* < 0.05). There was no significant difference in Duv (Supplementary Table S1).

Reproducibility analysis showed that the intraclass correlation coefficients of inter-observer and intra-observer were 0.936 and 0.918 for Duv, 0.888 and 0.880 for Vmax, 0.849 and 0.864 for UAPI, 0.911 and 0.881 for Quv, and 0.880 and 0.825 for VAI, respectively (Table 3).

**Table 3:** Interclass correlation coefficients for Doppler measurements.

	Intraobserver		Interobserver	
	ICC	95%CI	ICC	95%CI
Duv	0.918	0.767–0.963	0.936	0.798–0.973
Vmax	0.880	0.798–0.930	0.888	0.810–0.935
UAPI	0.864	0.773–0.921	0.849	0.748–0.911
Quv	0.881	0.773–0.936	0.911	0.842–0.950
VAI	0.825	0.693–0.900	0.880	0.790–0.932

ICC, interclass correlation coefficient; CI, confidence interval; Duv, diameter of umbilical vein; Quv, umbilical vein blood flow volume, UAPI, umbilical artery pulsatility index; VAI, venous–arterial index.

## Discussion

Previous studies have explored the predictive power of VAI in the development of intrapartum fetal distress or fetal outcomes, but the sample size is usually small. This study found that VAI decreased significantly in the compromised groups and exhibited better diagnostic performance in predicting compromised neonates than Quv and UAPI in a large sample. In addition, different from umbilical venous blood flow, VAI presented biphasic changes and reached its peak value at 32 weeks of gestation in fetuses with normal outcome.

Our results showed that Duv, Vmax and Quv increased with GA, while UAPI decreased as pregnancy proceeded under normal circumstances. The alterations in Duv, Vmax, Quv and UAPI were consistent with previous studies on umbilical vein blood flow (1, 11, 13). Although the placental blood flow also increased with fetal body weight, the nQuv declined slightly with gestational age. Martin et al. exhibited a negative correlation between nQuv and GA from 27 weeks to term (5). G. Acharya et al. demonstrated an increase in UV blood flow normalized for fetal weight from 19 to 25 weeks followed by a decrease after 25 weeks (13). The correlation between nQuv and GA presented in our study is also similar to their experiments. The increased Duv, Vmax and fetal body weight during pregnancy might explain the constancy of the placental blood flow per unit (5). Our study showed that the values of VAI showed biphasic changes. It increased significantly from 24 to 32 weeks and declined gradually after 32 weeks. The blood circulation of the fetus is redistributed during normal pregnancy, including increased blood flow in the internal organs and lower limbs (14). Due to the increased number of small vessels in the placental villi, the UAPI decreased with GA indicating a reduction in arterial resistance. Since VAI is calculated as the combination of nQuv and UAPI, it presented an upward trend first and then a downward trend in the second and third trimesters. Our study showed that the VAI reached its peak value at the 32nd week of gestation.

Our study found that Quv was significantly lower in the poor outcome group than in the controls, while UAPI increased obviously. Previous studies have already reported decreased umbilical venous volume flow in IUGR fetuses (1, 2, 15, 16). Our study demonstrated that Quv decreased significantly in the fetuses with poor outcomes. This is similar to the study of Rigano et al. They suggested that this reduction in blood flow is due to reduced umbilical vein velocity. As the VAI was calculated as the ratio of nQuv and UAPI, the compromised group exhibited a significantly lower VAI than the controls. In this study, VAI presented better diagnostic performance than Quv and UAPI. The ROC curve showed that the sensitivity and specificity of the VAI to predict a compromised neonate were 95.15% and 99.4%, respectively, at a cutoff value of 120 ml/min/kg. We presume this cutoff as the warning value of the VAI for predicting fetal outcome in middle-late pregnancy. Tchirikov M et al. suggested a cutoff of 100 ml/min/kg for the VAI as a warning value, which is lower than the value we obtained (17). As both studies choose the intra-abdominal segment of the umbilical cord to measure Quv, the difference may be due to the sample size and the distribution of gestational age. The warning value indicates that the fetus has chronic hypoxia but no serious intrauterine restriction at the time of the calculation. As a combination of the blood flow volume rate in the umbilical vein and the UAPI, VAI might be provided as an additional Doppler parameter in clinical setting.

Our results showed significant changes in Quv, UAPI and VAI in the compromised group. However, the indexes of outcome score in our study included indexes of both chronic impairment (birth weight centile, gestational age at birth) and acute impairment (PH at delivery, Apgar score at 1 min and respiratory support). We further divided the compromised group into fetuses of birth weight ≥10th percentile and of birth weight <10th percentile. The Quv and VAI was also significant decreased in the group of birth weight <10th percentile than that in the group of birth weight ≥10th percentile. Chronic impairment would be affected by placental flow alterations, while the other indexes might also be influenced by other factors during labor or neonatal care that could be acutely responsible of a neonatal adaptation besides umbilical venous and arterial flow. In this study, only 9.7% of neonates were born spontaneously in the compromised group, while 83.6% of the neonates were born spontaneously in controls. Therefore, the mode of delivery may not improve the statistical difference of Doppler parameters in the two groups. It is still necessary to incorporate other elements during neonatal care to further invalidate the value of VAI for predicting the fetal outcome.

This study also has limitations that should be highlighted. First, the accurate estimation of Quv needs to be considered. To minimize the impact of fetal position and small gestational age, Vmax and Duv were measured following the “maximum principle”. Although both the free-loop and the intra-abdominal portion of the umbilical cord have been used for measurement of Quv, a recent study demonstrated that the variance of Quv measurement at the free loop became large as the measured value increased, while the variance at the intra-abdominal portion was not influenced by the measured value (10). Second, as this is a cross-sectional study, all results were based on a single middle-late trimester observation. Longitudinal studies are needed to further validate the alterations.

In conclusion, the umbilical VAI of the compromised group was significantly lower than that of the control group. VAI presents better diagnostic performance in the prediction of perinatal outcome in the second half of pregnancy. A cutoff value of 120 ml/min/kg might be used as the warning value for predicting the fetal outcome.

## Data Availability

The raw data supporting the conclusions of this article will be made available by the authors, without undue reservation.
