# Supplementary material for: The abnormal umbilical venous–arterial index in the second half of pregnancy is associated with fetal outcome: A retrospective cross-sectional study
Source: Front Pediatr. 2023 Mar 10;11:1036359. doi: 10.3389/fped.2023.1036359 (PMC10036777; doi:10.3389/fped.2023.1036359)
Supplement: Supplementary file 1 [file Table1.docx]

Supplementary Table 1 Clinical and Doppler parameters of controls and fetuses with compromised outcome divided into the groups of birth weight ≥10th percentile and <10th percentile.

| Parameters | Control group  (n=730) | Compromised group (n=103) | | P value  (Birth weight percentile ≥10th versus <10th) |
| --- | --- | --- | --- | --- |
|  |  | Birth weight (percentile) ≥10th (n=77) | Birth weight (percentile) <10th (n=26) |  |
| GA at scan | 32(28-36) | 31.3(29.6-31.9) | 31.3(27.5-32.5) | 0.665 |
| Duv,cm | 0.59(0.51-0.66) | 0.54(0.45-0.61) | 0.54(0.44-0.55) | 0.396 |
| Vmax, cm/s | 33.6(28.8-38.3) | 17.7(15.9-19.9) | 15.9(13.2-18.1) | 0.007 |
| Quv, ml/min | 271.2(188.9-361.3) | 136.4(73.9-155.3) | 103.1(68.67-139.7) | 0.049 |
| nQuv, ml/min/kg | 148.6(131.8-167.8) | 83.8(71.2-98.8) | 80.1(65.8-91.7) | 0.108 |
| UAPI | 0.8(0.7-0.9) | 1.0(0.9-1.1) | 1.0(1.0-1.2) | 0.019 |
| VAI, ml/min/kg | 184.8(163.6-201.4) | 86.8(75.8-98.8) | 76.5(58.6-85.7) | 0.003 |

GA, gestational age; Duv, diameter of umbilical vein; Quv, umbilical vein volume blood flow, nQuv, Normalized umbilical vein blood flow volume; UAPI, umbilical artery pulsatility index; VAI，venous–arterial index.
