# Supplementary figures and images for: The abnormal umbilical venous–arterial index in the second half of pregnancy is associated with fetal outcome: A retrospective cross-sectional study
Source: Front Pediatr. 2023 Mar 10;11:1036359. doi: 10.3389/fped.2023.1036359 (PMC10036777; doi:10.3389/fped.2023.1036359)

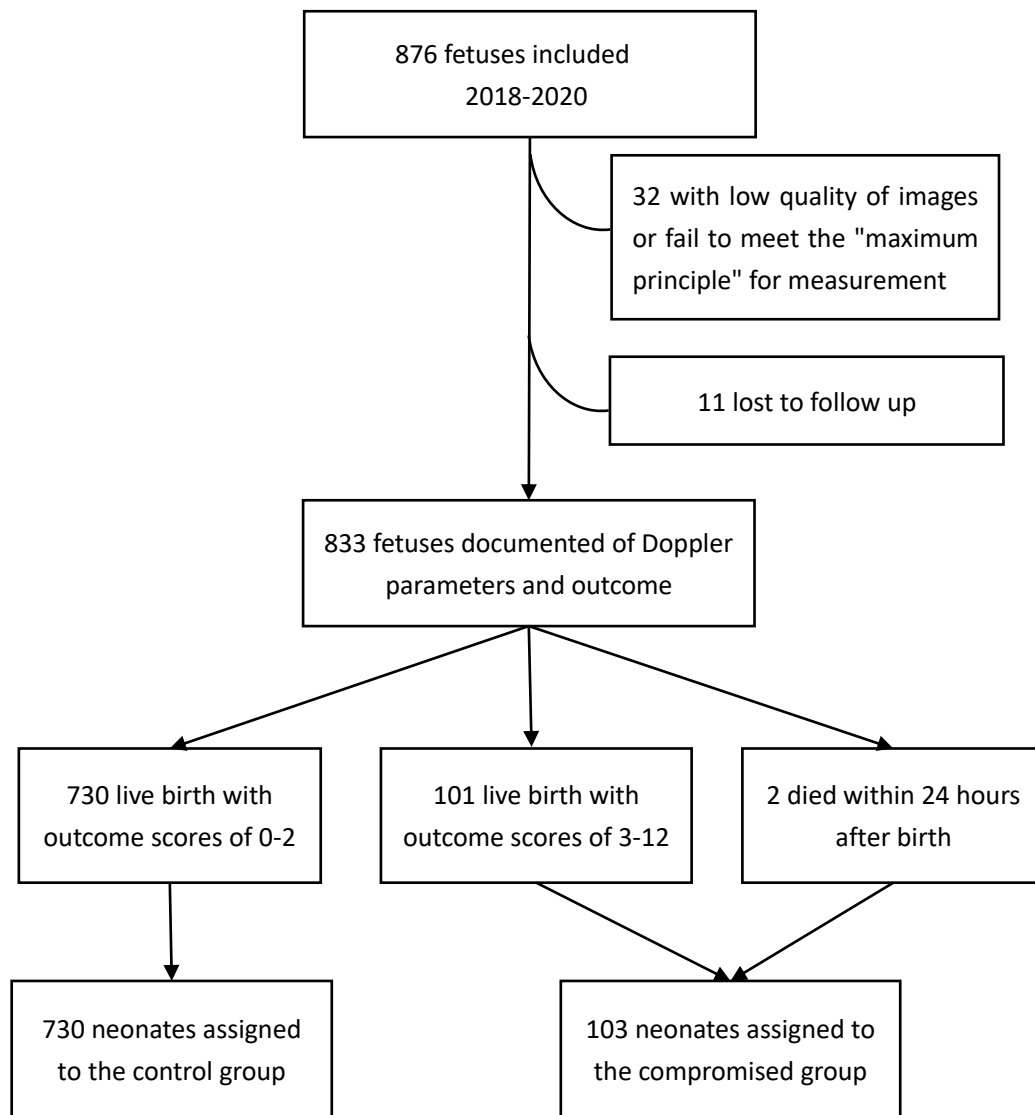

Supplementary Figure 1. Flow chart of study population

Supplement: Supplementary file 2 [file Datasheet1.pdf]
